# T cell mediated immunity after combination therapy with intralesional PV-10 and blockade of the PD-1/PD-L1 pathway in a murine melanoma model

**DOI:** 10.1371/journal.pone.0196033

**Published:** 2018-04-25

**Authors:** Hao Liu, Amy Weber, Jennifer Morse, Krithika Kodumudi, Ellen Scott, John Mullinax, Amod A. Sarnaik, Shari Pilon-Thomas

**Affiliations:** 1 Department of Immunology, H Lee Moffitt Cancer Center and Research Institute, Tampa, Florida, United States of America; 2 Sarcoma Program, H Lee Moffitt Cancer Center and Research Institute, Tampa, Florida, United States of America; 3 Department of Cutaneous Oncology at H. Lee Moffitt Cancer and Research Institute Tampa, Florida, United States of America; Mie University Graduate School of Medicine, JAPAN

## Abstract

Intralesional (IL) injection of Rose Bengal (PV-10) induces regression of injected and uninjected lesions in several murine tumor models. In this study, we investigated the anti-tumor response of combining IL PV-10 with blockade of the PD-1 / PD-L1 pathway and the role of immune cell populations in eliciting this response. To investigate the role of T cell subsets in mediating an immune response, B16 or M05 melanoma-bearing mice received combination therapy as well as CD8^+^, CD4^+^, or CD25^+^ depleting antibodies. Tumor growth was measured. T cells were collected from spleens or tumors, and phenotype, activation markers, and reactivity were measured. Splenocytes from mice treated with combination therapy had increased OVA antigen-specific CD8^+^ T cells in M05-tumor-bearing mice. Depletion of CD4^+^ T cells or regulatory T cells (Tregs) in combination with IL PV-10 and anti-PD-1 antibody treatment resulted in an enhanced anti-tumor effect. Treatment with CD8^+^ depleting antibody abrogated anti-tumor immunity. These results support a clinical study for the safety and anti-tumor immune responses with combination therapy of IL PV-10 and PD-1/PD-L1 blockade.

## Introduction

Rose bengal disodium (RB) is a xanthene dye previously used as a diagnostic for liver function and currently as an agent to detect injury within the eye [1]. PV-10 is a 10% RB solution for intralesional (IL) injection into tumors. After injection, PV-10 localizes in the lysosomes of tumor cells and causes lysis [2]. Release of High Mobility Group Box 1 (HMGB1) from the necrotic tumor cells can activate tumor-resident dendritic cells resulting in the induction of a tumor-specific T cell response [3]. In tumor-bearing mice, IL PV-10 therapy induces the regression of both injected and untreated tumor lesions [4].

In melanoma patients, treatment with IL PV-10 also results in increased serum HMGB1 levels and improved anti-tumor activity of circulating CD8+ T cells [3]. In patients with melanoma, a 48% overall response (OR) in treated lesions and a 27% OR in uninjected lesions was measured after IL PV-10 injection [5]. A follow-up phase 2 study has demonstrated a similar response rate in treated and uninjected lesions [6]. PV-10 is currently under investigation in a phase 3 clinical trial as a single agent vs. chemotherapy or oncolytic viral therapy in locally advanced melanoma patients (NCT 02288897).

The lack of adequate co-stimulation and the presence of inhibitory factors can lead to T cell dysfunction in the tumor microenvironment. Activated T cells at the tumor site express multiple checkpoint (co-inhibitory) molecules including cytotoxic T-lymphocyte associated protein-4 (CTLA4), programmed cell death 1 (PD-1), lymphocyte-activation gene 3 (Lag3), T cell immunoglobulin mucin 3 (TIM3), and B and T lymphocyte attenuator (BTLA). Ligation of these checkpoint receptors at the tumor site leads to T cell exhaustion and downregulation of effector functions. Blockade of these receptors can restore T cell activation and function leading to improved anti-tumor immunity [7].

CTLA4 serves as the dominant “off” switch for T cell responses. Immunotherapy using ipilimumab, a CTLA4 blocking antibody, has improved overall survival of patients with metastatic melanoma [8]. PD-1 is upregulated on both activated and exhausted T cells and restricts T cell function. The interaction of PD-1 with its ligand PD-L1 or PD-L2 has emerged as a dominant influence in T cell suppression at the tumor site. Blockade of this PD-1/PD-L1 or PD-L2 interaction with anti-PD-1 antibody treatment enhances T cell migration into tumors [9]. Treatment with an anti-PD-1 antibody has been studied in clinical trials and has resulted in a 31–40% response rate and 62–73% 1 year overall survival rate in metastatic cutaneous melanoma patients when used as monotherapy [10–12].

In this study, we investigated combination therapy of IL PV-10 with blockade of PD-1 and PD-L1 in a murine melanoma model, and we have examined the role played by specific immune T cell populations in eliciting a tumor specific response.

## Material and methods

### Animals

C57BL/6 mice were purchased from Charles River Laboratories (Indianapolis IN). Mice were kept at the Comparative Medicine Facility at the Moffitt Cancer Center. Mice underwent daily observation and were euthanized when a single subcutaneous tumor exceeded 200 mm^2^. Mice were euthanized humanely according to the American Veterinary Medical Association Guidelines, using CO_2_ inhalation. Animal experiments were approved by the Institutional Animal Care and Use Committee at the University of South Florida. The University of South Florida Comparative Medicine is fully accredited by AAALAC International (#000434), has an assurance #A4100-01 on file with OLAW/PHS and maintains registration #58-R-0015 with USDA/APHIS/AC.

### Cell lines and cell culture

The B16 melanoma cell line was purchased from the American Type Culture Collection (ATCC). Cells were cultured in RPMI as previously described [13]. The OVA-expressing M05 melanoma cell line [14] was cultured in RPMI with 0.8 mg/ml G418 (neomycin). After thawing, cell lines were passaged a maximum of 10 times and routinely tested negative for mycoplasma contamination.

### Combination therapy

Provectus Biopharmaceuticals, Inc. (Knoxville, TN) provided the PV-10 used in these studies. For murine experiments, 1x10^5^ B16 cells or 3x10^5^ M05 tumor cells were injected SC into one or both flanks of C57BL/6 mice. On day 7–13 after tumor implantation, PV-10 or PBS was injected IL followed the next day by intraperitoneal (IP) injection of 300 μg of anti-PD-1 antibody (clone RMP1-14) or anti-PD-L1 antibody (clone 10F.9G2). Additional experiments used IP injection of 300 μg of anti-CTLA-4 antibody (clone 9H10). Control antibodies included normal rat IgG (NrIgG). For depletion experiments, M05 tumor-bearing mice were given either 300 μg of NrIgG antibodies as a control, anti-CD8 antibody (clone 2.43), anti-CD4 antibody (clone GK1.5), or anti-CD25 antibody (clone PC61) to deplete T cell subsets starting 3 days prior to M05 injection. Antibodies were given twice per week until the completion of the experiment. All antibodies were purchased from BioXcell (West Lebanon, NH).

### Flow cytometry and tetramer staining

Spleens and tumors were processed as previously described [13]. Cells were then stained with anti-mouse CD8, CD4, and CD3 antibodies (all from BD Bioscience, San Diego, CA) or with H-2 K^b^ /SIINFEKL tetramer (MBL international, Woburn, MA) for analysis by flow cytometry. Dead cells were excluded as previously described [13]. Data were acquired by an LSR II flow cytometer equipped with five lasers (BD Biosciences), and FlowJo software was used for analysis (Tree Star, Ashland, OR).

### Assessment of IFN-gamma by ELISA

To analyze murine samples for the presence of IFN-gamma, mice received 3x10^5^ M05 cells SC on Day 0. Spleens were harvested on Days 21–24 after tumor implantation. Supernatants were collected after a 48 hour co-culture of splenocytes with M05 or irrelevant cells. IFN-gamma was measured by ELISA (BD Bioscience) according to the protocol supplied by the manufacturer.

### Statistical analysis

For comparison of *in vitro* measurements, a one-way ANOVA (followed by Tukey post hoc test) was performed. For comparison of *in vivo* measurements, the same test was performed using tumor measurement taken at each time point. A Mann-Whitney test was used to compare between two treatment groups. All statistical analysis were performed using GraphPad Prism software. Data with a p value less than 0.05 was deemed to be statistically significant.

## Results

### Expression of PD-L1 on M05 melanoma and expression of PD-1 on CD8^+^ T cells infiltrating M05

**Fig 1:**
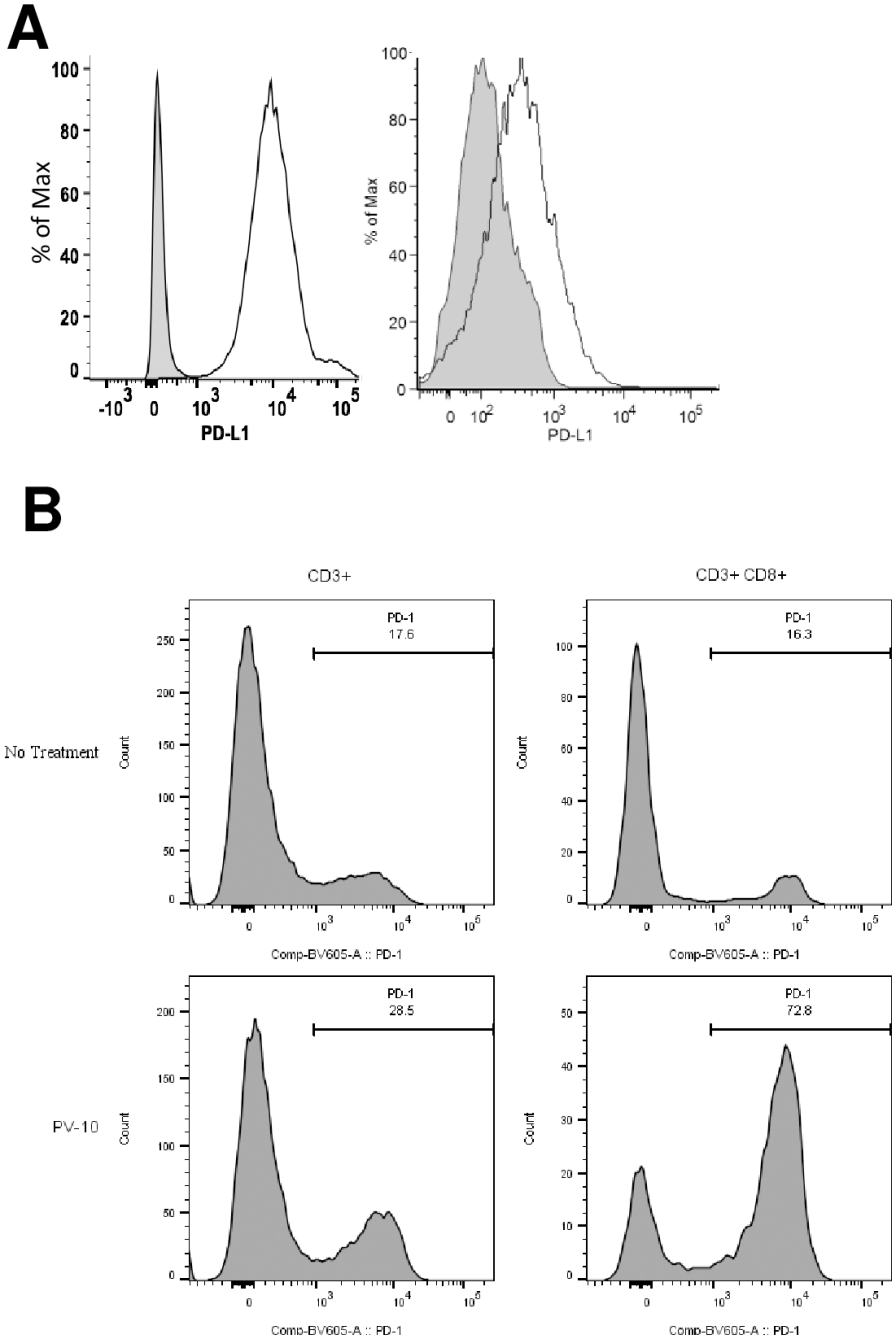
Expression of PD-L1/PD-1 in an M05 melanoma model. (A) Expression of PD-L1 on M05 tumor cells after (left) no treatment or (right) IL PV-10 injection as measured by flow cytometry. Gray histogram: isotype control. (B) Expression of PD-1 on total CD3^+^ and CD3^+^CD8^+^ tumor infiltrating lymphocytes (TIL) isolated from untreated or IL PV-10 treated M05 tumors.

We first wanted to determine whether blockade of the PD-L1/PD-1 pathway was feasible in this model. We measured the expression of PD-L1 on freshly isolated M05 tumors and PD-1 on tumor infiltrating T cells. Tumors were collected from C57BL/6 mice that had been untreated or treated with IL PV-10. As shown in Fig 1A, M05 tumors from untreated mice express PD-L1 (left panel). PD-L1 is downregulated in M05 tumors treated with IL PV-10 but is still expressed (right panel). Using flow cytometry, we measured the expression of PD-1 on CD3^+^ and CD8^+^ T cells in digested tumors (Fig 1B). We found that PD-1 is expressed on CD8^+^ T cells infiltrating PD-L1-expressing M05 tumors and is upregulated on CD8^+^ T cells that infiltrate tumors treated with IL PV-10.

### Combination therapy with IL PV-10 and PD-1 blockade delays tumor growth

**Fig 2:**
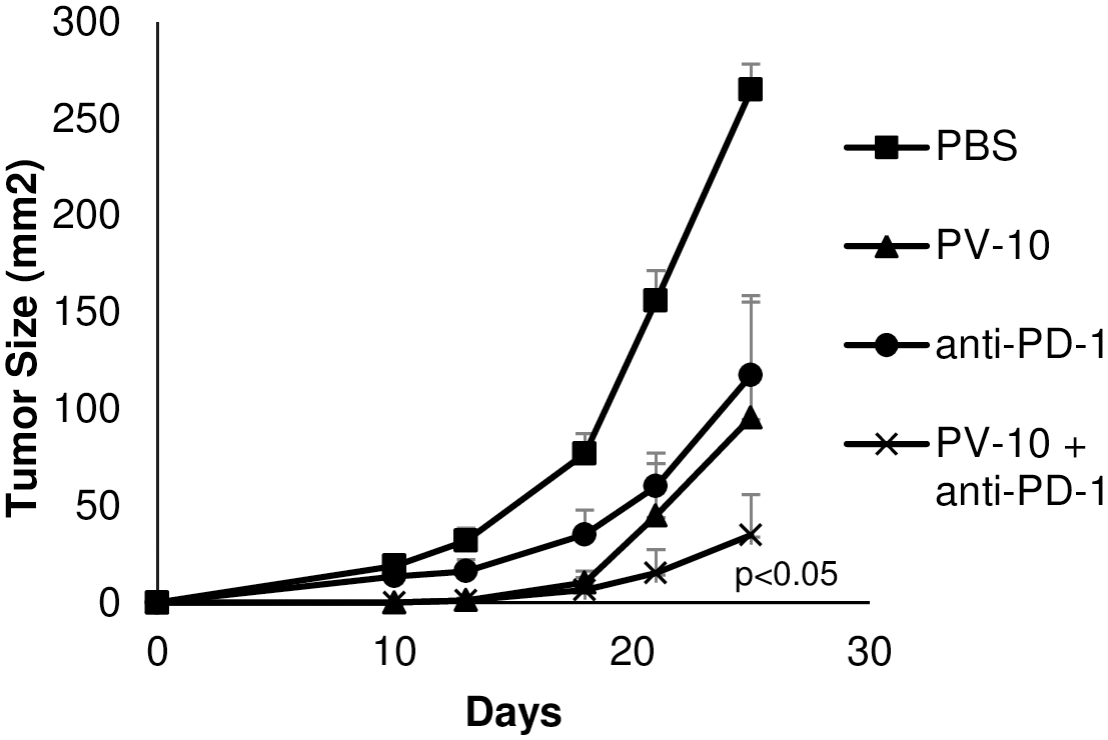
Combination therapy with IL PV-10 and anti-PD-1 results in delayed tumor growth. Mice received 3x10^5^ M05 tumor cells SC on a single flank on Day 0. On Day 7, mice received either 50 μl PV-10 or PBS IL, and mice received anti-PD-1 antibody IP twice per week beginning on Day 8. Tumors were measured until the endpoint was reached.

Next, we measured whether IL injection of PV-10 alone or in combination with anti-PD-1 antibody therapy led to delayed tumor growth in M05 tumor-bearing mice. Mice received 3x10^5^ M05 tumor cells SC on a single flank on Day 0. On Day 7, mice received a single IL injection (50 μl PV-10 or PBS) followed by IP injection of anti-PD-1 antibodies on Day 8. Antibody therapy was continued twice per week for 2–3 weeks. As shown in Fig 2, injection of either PV-10 or anti-PD-1 antibodies led to delayed tumor growth. Combination therapy with PV-10 and PD-1 antibody blockade further delayed growth of M05 tumors (p<0.05 compared to all other groups).

### Increased M05- specific T cell activity after IL PV-10 injection and PD-1 blockade

**Fig 3:**
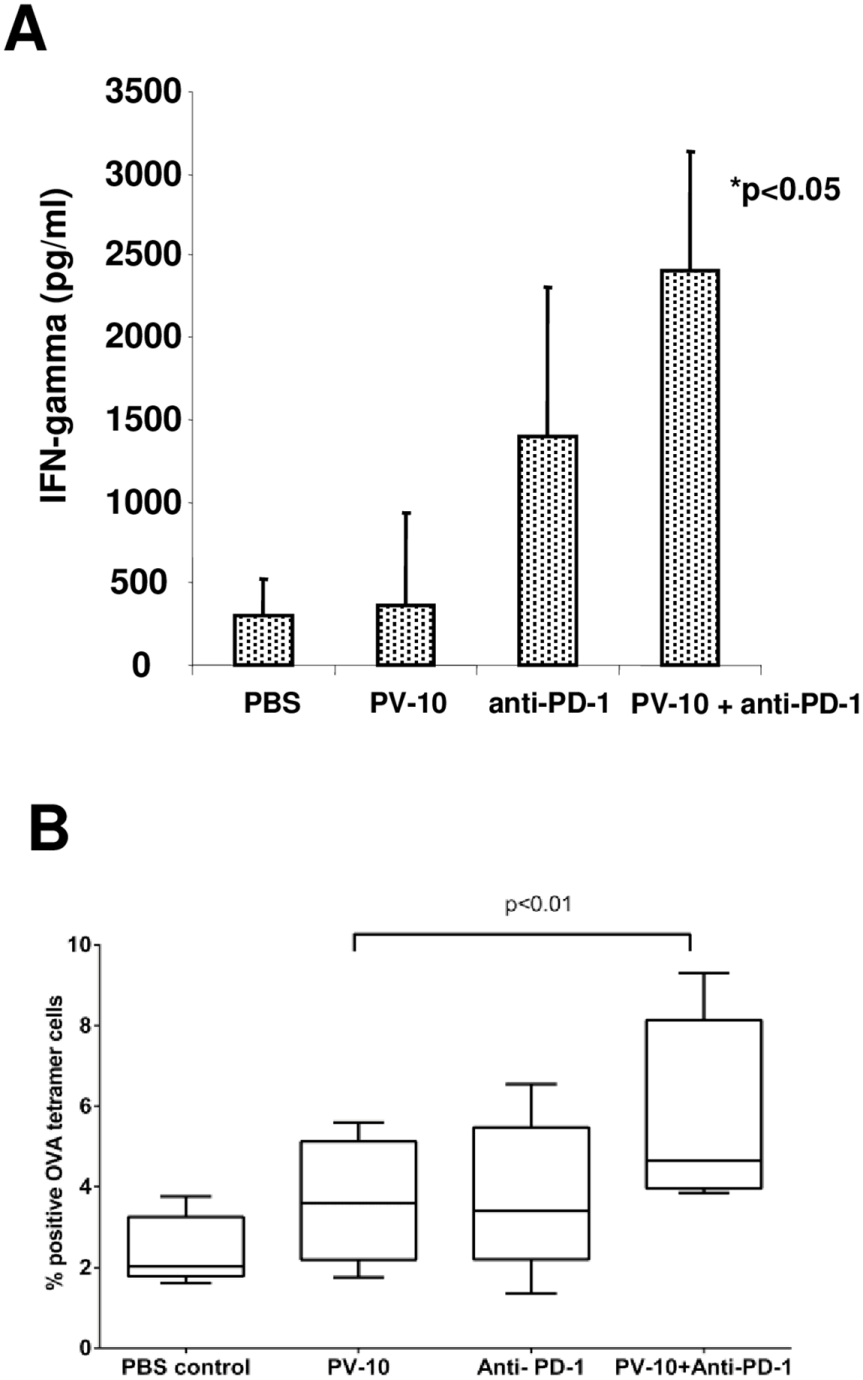
Increased M05-specific T cell activity after IL PV-10 injection and PD-1 blockade. Mice received 3x10^5^ M05 tumor cells SC on a single flank on Day 0. On Day 7, mice received 50 μl PV-10 or PBS IL, and mice received anti-PD-1 antibody IP twice per week beginning on Day 8. Spleens were harvested on Day 21–24. (A) Splenocytes were co-cultured with M05 cells for 48 hours and supernatants were collected. IFN-gamma was measured by ELISA. (B) CD8^+^ and OVA-tetramer positive T cells were measured by flow cytometry.

We next evaluated whether combination therapy with IL PV-10 and blockade of PD-1 led to a systemic anti-tumor immune response. Mice received 3x10^5^ M05 tumor cells SC on a single flank on Day 0. On Day 7, mice received a single IL injection (50 μl PV-10 or PBS) followed by anti-PD-1 antibody IP twice per week beginning on Day 8. Spleens were harvested on Day 21–24 after tumor implantation. Splenocytes were co-cultured with M05 cells for 48 hours, then supernatants were collected and IFN-gamma was measured by ELISA. As OVA is a foreign antigen in these mice, IL injection of PBS alone also elicited production of IFN-gamma in response to M05 tumor. Splenocytes collected from mice that received combination therapy with IL PV-10 and PD-1 blockade demonstrated increased IFN-gamma secretion in response to M05 cells (Fig 3A, p<0.05 compared to PBS, PV-10 alone, or anti-PD-1 antibody alone). We also measured whether OVA-specific T cell responses were increased in the splenocytes of M05 bearing mice. As shown in Fig 3B, M05-bearing mice treated with IL PV-10 and anti-PD-1 antibodies had an increase in the percentage of CD8+ and OVA-tetramer positive CD8^+^ T cells (p<0.01 compared to treatment with IL PV-10 alone).

### IL PV-10 or PD-1 blockade increases TIL infiltration into tumors

We evaluated the infiltration of tumor-specific tumor infiltrating lymphocytes (TIL) into bystander tumors in mice treated with IL PV-10. Mice received M05 tumor cells SC on each flank on Day 0. On Day 13, mice were injected IL with 50 μl PV-10 or PBS in the right flank tumor, followed by IV injection of violet-labelled CD45.1^+^ OT-I T cells and IP injection of anti-PD-1 antibodies. On Day 17, tumors on the left flank were harvested. Digested tumors were stained for CD45.1 and analyzed by flow cytometry (S1A Fig). Increased numbers of transferred T cells were measured in all treated groups (p<0.01 compared to PBS treated) but no difference was measured between mice treated with the combination compared to IL PV-10 or anti-PD-1 antibodies alone. Cell viability of the bystander tumor was also analyzed by flow cytometry (S1B Fig). While treatment with anti-PD-1 antibodies alone had no effect, bystander tumors in mice treated with IL PV-10 alone or in combination anti-PD-1 antibodies had fewer viable cells (p<0.05 compared to treatment with anti-PD-1 antibodies alone).

### PV-10 injection increases M05-specific response in bystander tumor

We also measured the activity of T cells isolated from bystander tumors after treatment with IL PV-10 in combination with anti-PD-1 antibodies. Mice were injected with M05 cells SC in both flanks on Day 0. On Day 10, mice received 50 μl PBS or PV-10 IL in the left flank. Mice received NrIgG or anti-PD-1 antibodies IP on Days 10 and 13. On Day 17, the un-injected bystander lesion was resected, and TIL were isolated. TIL were co-cultured with M05 or irrelevant MC38 tumor cells for 48 hours and supernatants were collected. IFN-gamma was measured by ELISA. As shown in S2 Fig, treatment with anti-PD-1 antibodies alone, IL PV-10 alone, or combination therapy resulted in increased IFN-gamma production on co-culture with M05 cells compared to control mice.

### Effect of combination therapy with IL PV-10 and PD-1 blockade is mediated by CD8^+^ T cells

**Fig 4:**
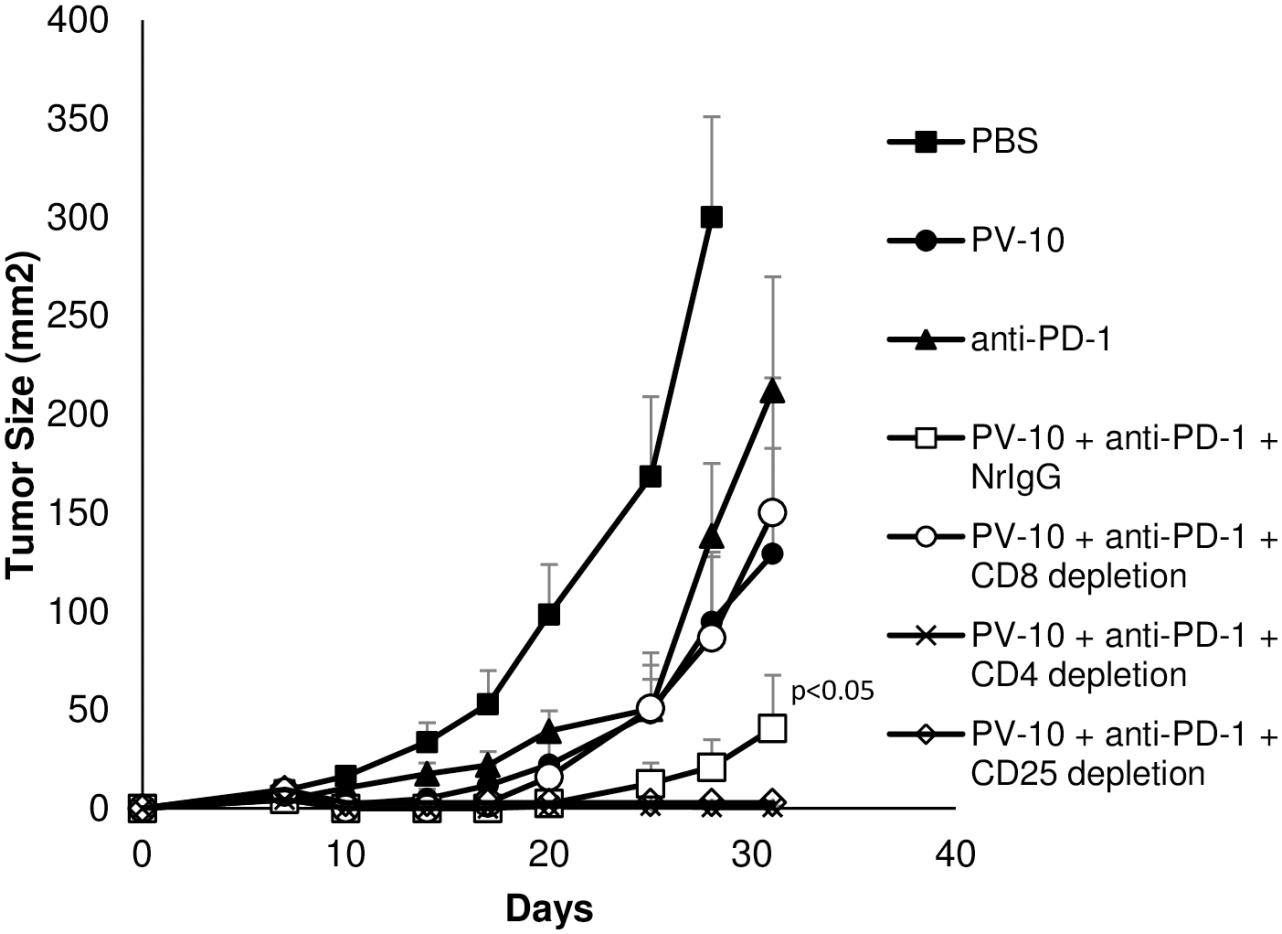
Effect of combination therapy with IL PV-10 and PD-1 blockade is mediated by CD8^+^ T cells. Mice received 3x10^5^ M05 tumor cells SC on a single flank on Day 0, and were given 300 μg IP NrIgG control antibodies, 2.43 antibody to deplete CD8^+^ T cells, GK1.5 antibody to deplete CD4^+^ T cells, or PC61 antibody to deplete CD25^+^ Tregs. Antibodies were given twice per week until the completion of the experiment. On Day 7, mice received 50 μl PV-10 or PBS IL, and mice received anti-PD-1 antibody IP twice per week beginning on Day 8.

To determine the role of T cell subsets in mediating tumor regression of M05 tumors, mice were treated with combination IL PV-10 and anti-PD-1 antibodies alone or in the presence of T cell depleting antibodies. Control mice were untreated, treated with anti-PD-1 antibody alone, or IL PV-10 alone. Depletion of T cell subsets was verified (S3 Fig). As shown in Fig 4, treatment with combination IL PV-10 and anti-PD-1 significantly delayed tumor growth compared to single treatments alone (p<0.05). Depletion of CD8^+^ T cells led to the loss of anti-tumor effects mediated by the combination therapy, whereas depletion of CD4^+^ T cells led to a decrease in tumor growth compared to mice treated with IL PV-10 in combination with anti-PD-1 antibodies and control NrIgG. To further investigate the role of CD4^+^ T cells, M05 tumor-bearing mice treated with IL PV-10 and anti-PD-1 antibodies received antibodies to deplete CD25^+^ regulatory T cells (Tregs). Depletion of Tregs was equally as effective as CD4^+^ T cell depletion at enhancing the anti-tumor immunity induced by combination therapy with IL PV-10 and anti-PD-1 antibodies (p<0.05 compared to all other treatment groups).

### IL PV-10 in combination with anti-PD-1 antibody delays the growth of treated and bystander M05 tumors

**Fig 5:**
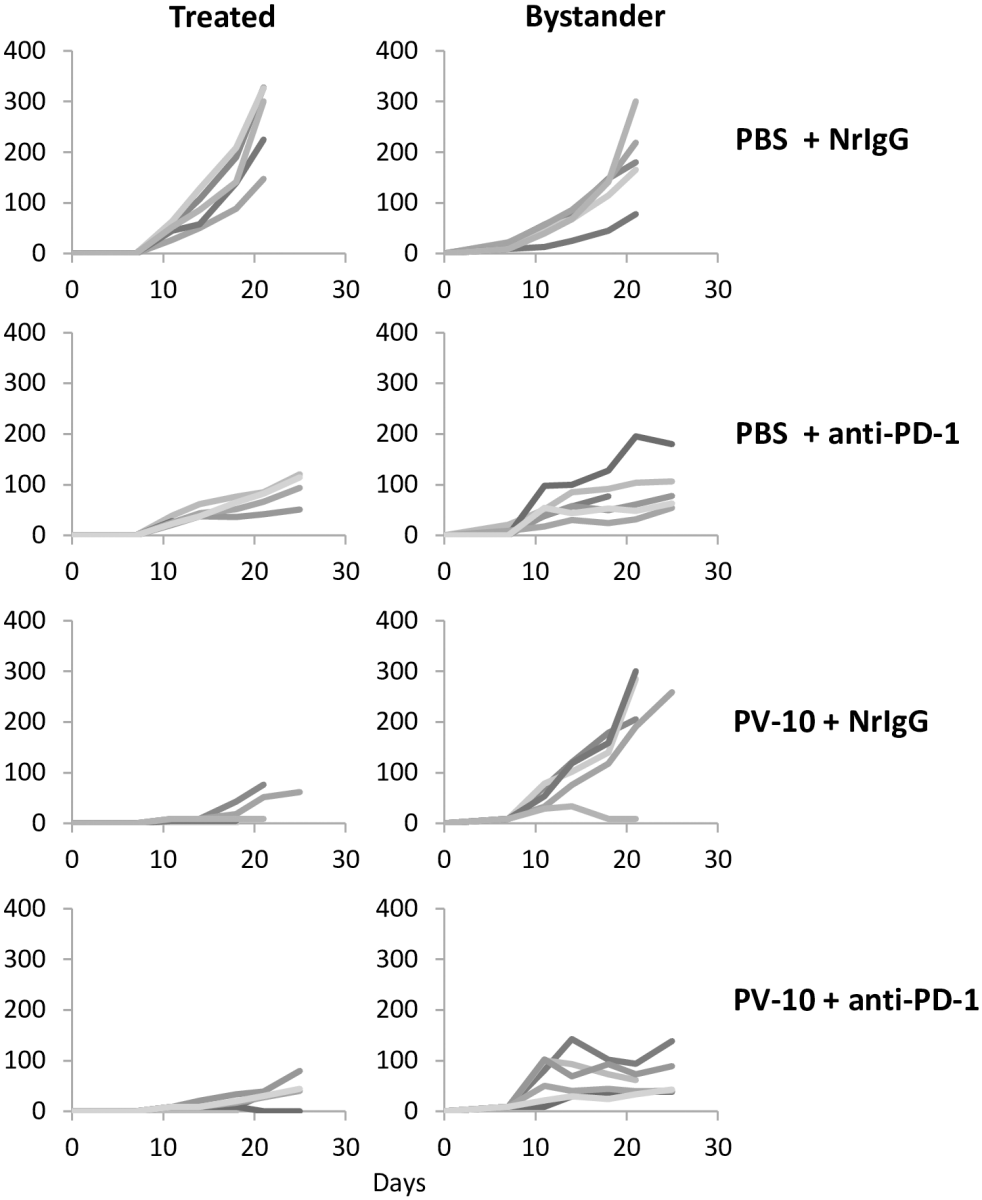
IL therapy with PV-10 in combination with anti-PD-1 antibodies leads to delayed growth of bystander M05 tumors. Mice received 3x10^5^ M05 tumor cells SC on bilateral flanks on Day 0. On Day 7, right flank tumors were injected with 50 μl PV-10 or PBS IL. Mice received 300 μg anti-PD-1 and NrIgG antibodies beginning on Day 8 and continuing 2x/week until mice had reached endpoint. Tumor growth was measured in (left panel) tumors treated with IL PBS or PV-10 and (right panel) untreated, bystander tumors. Each line represents the growth of tumor in a single mouse (n = 5–6 mice per treatment group).

Mice received M05 tumor cells SC on bilateral flanks on Day 0 to evaluate the growth of treated and bystander melanoma lesions. On Day 7, 50 μl PBS or PV-10 was injected IL into the tumor on the right flank. Mice received NrIgG control or anti-PD-1 antibodies beginning on Day 8 and continuing twice per week until the end of the experiment. While treatment with IL PV-10 alone or in combination with anti-PD-1 antibody led to a delay in growth of treated tumor, only the combination therapy was able to delay the growth of uninjected bystander tumor (Fig 5, p<0.05 compared to PBS and PV-10, p = 0.06 compared to anti-PD-1 alone).

### Increased anti-tumor immunity after IL PV-10 in combination with checkpoint antibodies delays growth of treated B16 tumors

We also measured the effect of targeting checkpoint receptors on the growth of B16 melanoma tumors that do not express the foreign OVA antigen. Mice received B16 tumor SC on Day 0. Starting on Day 10, mice received one injection of PV-10 IL, followed by IP injections of anti-CTLA-4, anti-PD-1, or anti-PD-L1 antibodies twice a week for 2 weeks. Combination therapy with IL PV-10 and checkpoint antibodies led to a delay in B16 tumor growth (S4 Fig, p<0.05 for each combination therapy compared to IL PV-10 alone). As combination therapy with IL PV-10 and anti-PD-L1 was associated with the most delayed growth of B16 tumor, this combination was chosen for further experiments.

### IL PV-10 in combination with anti-PD-L1 antibody delays growth of treated and bystander B16 tumors

**Fig 6:**
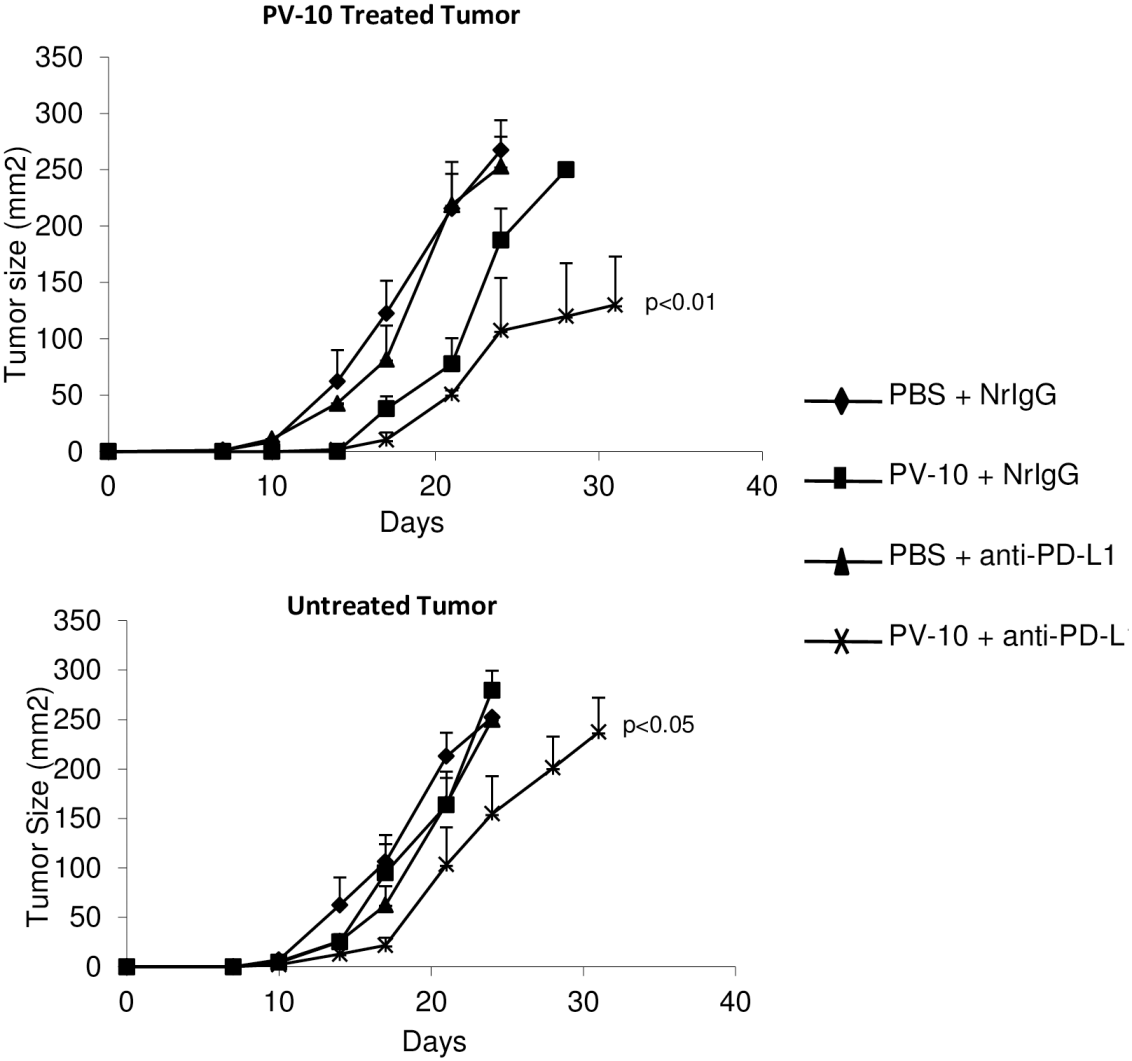
IL therapy with PV-10 in combination with anti-PD-L1 antibodies leads to delayed growth of treated and bystander B16 tumors. Mice received 1x10^5^ B16 tumor cells SC on bilateral flanks on Day 0. On Day 7, right flank tumors were injected with 50 μl PV-10 or PBS IL. Mice received 300 μg anti-PD-L1 or NrIgG antibodies beginning on Day 8 and continuing 2x/week until mice had reached endpoint. Combination of PV-10 and anti-PD-L1 led to a delayed tumor growth in A) B16 tumors treated with IL PV-10 and B) untreated B16 bystander tumors.

**Fig 7:**
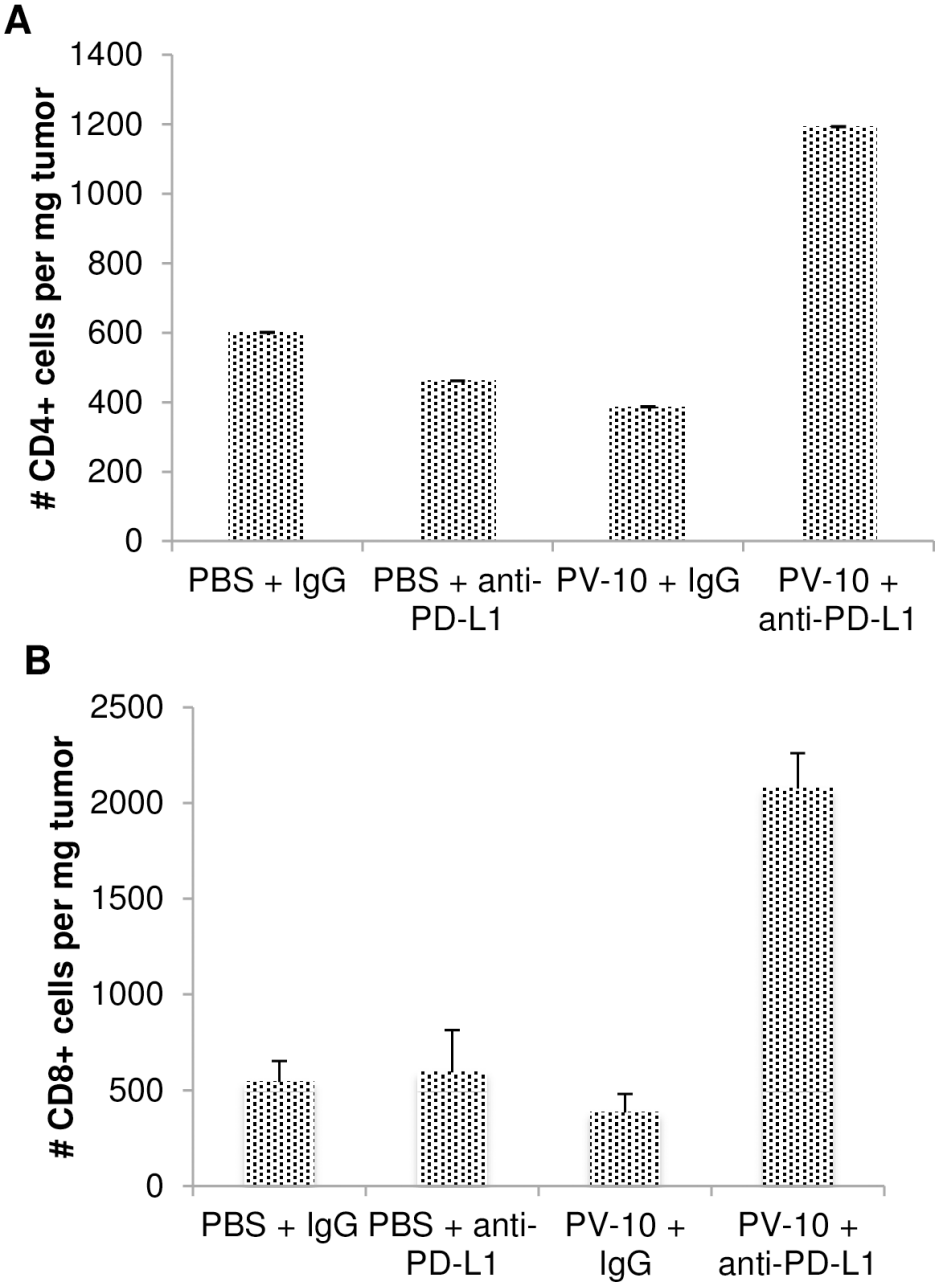
Increased T cell infiltration into Bystander M05 Tumor after IL PV-10 injection and anti-PD-L1 antibody treatment. Mice received 3x10^5^ M05 tumor cells SC on a both flanks on Day 0. On Day 7, mice received 50 μl PV-10 or PBS IL, and mice received anti-PD-L1 or IgG antibody IP twice per week beginning on Day 8. Mice were euthanized on Day 21, tumors were harvested and TILs were isolated from tumors. Infiltrating (A) CD4^+^ and (B) CD8^+^ T cells were measured by flow cytometry.

We have previously shown that targeting PD-L1 in the B16 melanoma model can improve the anti-tumor T cell responses [15]. To measure regression of treated and bystander melanoma tumors, mice received B16 tumor cells SC on bilateral flanks on Day 0. On Day 7, mice were injected with 50 μl PBS or PV-10 IL in the tumor on the right flank. Mice received NrIgG or anti-PD-L1 antibodies beginning on Day 8 and continuing twice per week until the end of the experiment. While treatment with IL PV-10 alone or in combination with anti-PD-L1 antibody led to a delay in growth of treated tumor, only the combination therapy was able to delay the growth of uninjected bystander tumor (Fig 6). We evaluated the infiltration of T cell subsets into bystander tumors of treated mice. As shown in Fig 7, increased numbers of CD4^+^ and CD8^+^ T cells were measured in the bystander tumors of mice treated with IL PV-10 in combination with anti-PD-L1 antibodies (p<0.05 compared to all other treatment groups).

## Discussion

The incidence of melanoma has increased over the past 30 years, and it accounts for the majority of skin cancer deaths worldwide [16]. In patients with unresectable dermal or subcutaneous in-transit melanoma, intralesional (IL) therapy has shown great treatment potential [17]. IL therapy successfully induces both a local tumor ablation and systemic anti-tumor immunity in tumor-bearing mice, suggesting that the efficacy of this therapy could improve in combination with checkpoint inhibitors for metastatic melanoma patients.

Injection of PV-10 into tumor lesions has been shown to induce immunogenic cell death (ICD) in murine and human tumor cells. In murine models of colon cancer and melanoma, lysis of tumors by injection of IL PV-10 led to the release of HMGB1, dendritic cell (DC) maturation and migration into tumor-draining LNs, and the induction of CD8^+^ T cells that recognize tumor [3, 18]. In murine models of breast cancer and melanoma, IL PV-10 injection induced a systemic anti-tumor immune response that resulted in the rejection of both the injected and untreated lesions [4]. In patients with melanoma, injection of PV-10 resulted in a 50% objective response rate [6]. After one year, 8% of treated patients had no evidence of disease and complete regression of uninjected lesions were observed in 26% of these patients. Despite this success, there were few responders in patients with large numbers of uninjected lesions. Since it can be difficult to treat all tumors with IL therapy due to the number of lesions, combining IL therapy with therapies that activate and expand systemic anti-tumor T cell responses may lead to improved responses in metastatic patients [19].

Even when T cells are activated at a tumor site, the lack of adequate co-stimulation and presence of inhibitory factors can lead to T cell dysfunction. Activated T cells in the blood and at tumor sites express multiple checkpoints, including PD-1. PD-1 is upregulated on both activated and exhausted T cells and restricts T cell function. Blockade of PD-1 or its ligand PD-L1 augments the infiltration of T cells into tumors and reverses the exhaustion of T cells [9]. Antagonistic PD-1 antibodies are approved for the treatment of metastatic melanoma while PD-L1 antibodies are approved for a number of other indications.

In this study, IL PV-10 therapy in combination with blockade of the PD-1 pathway led to a reduction in tumor burden and an increase in tumor infiltrating lymphocytes. IL PV-10 has been shown to upregulate co-stimulatory markers on dendritic cells (DC) in tumor bearing mice [3]. As the CD28/B7 co-stimulatory pathway is crucial for effective therapy with PD-1 blockade, IL PV-10 may enhance the ability of DC to stimulate tumor-reactive T cells at the tumor site [20, 21]. Tumor regression was abrogated by depletion of CD8^+^ T cells, but depletion of CD4^+^ T cells improved the anti-tumor effects of IL PV-10 therapy. Combination therapy with IL PV-10, anti-PD-1 antibodies, and regulatory T cell (Treg) depletion led to complete regression of melanoma. This warrants further investigation into strategies to deplete Tregs, such as combination therapy with PV-10 and cyclophosphamide. Additionally, further investigation of the role of CD4^+^ T cells in this model is needed.

Blockade of PD-L1 in combination with IL PV-10 therapy also led to a delayed tumor growth in mice bearing B16 melanoma. While this combination did not enhance the production of IFN-gamma by CD8^+^ T cells in tumor-bearing mice beyond that of PD-L1 antibody alone (not shown), regression of uninjected bystander B16 tumor was measured, supporting the induction of a robust systemic anti-tumor response. While blockade of PD-1 or PD-L1 in combination with IL PV-10 therapy led to anti-tumor immunity in murine melanoma, many CD8^+^ T cells infiltrating tumors express other co-inhibitory molecules including Tim3, Lag3, CD160, CD244 and BTLA [22–25]. Additional studies to target these checkpoint receptors may lead to improved anti-tumor immunity when combined with IL injection of PV-10. In addition, strategies to target other regulatory subsets such as tumor-associated macrophages or myeloid derived suppressor cells may further enhance the induction of systemic anti-tumor immunity by IL PV-10. We have shown that combination therapy of IL PV-10 and PD-1 blockade leads to improved T cell infiltration into tumors. This combination therapy could possibly be exploited further to generate TIL products for use in adoptive cell therapy strategies.

The stimulation of tumor-specific immunity with IL PV-10 combined with systemic checkpoint inhibition may provide a path to improved outcome in patients with metastatic melanoma over that possible with either approach alone. In this study, we have shown the impact of combining systemic checkpoint blockade (PD-1, PD-L1) with the tumor-specific immune response induced by IL PV-10. Treatment with IL PV-10 and anti-PD-1 antibody resulted in a delay in tumor growth and enhanced T cell activation in the M05 tumor model. Similar effects were observed with IL PV-10 and anti-PD-L1 antibody in the B16 tumor model. The effect of combination therapy with IL PV-10 and PD-1 blockade is mediated by CD8^+^ T cells, and depletion of either CD4^+^ T cells or CD4^+^CD25^+^ Tregs enhances anti-tumor immunity in the M05 melanoma model. Together these results support further development of clinical trials to assess safety and anti-tumor T cell responses in patients after IL injection of PV-10 in combination with checkpoint blockade. A clinical study combining IL PV-10 and PD-1 blockade is currently ongoing (NCT 02557321).

## Supporting information

S1 Fig Increased TIL infiltration into treated tumor after IL PV-10 or PD-1 blockade. Mice received 3x10^5^ M05 tumor cells SC on Day 0. On Day 13, mice were injected IL with 50 μl PV-10 or PBS, 2.5x10^5^ violet-labelled CD45.1 OT-I T cells i.v., and 300 ug PD-1 IP. On Day 17, tumors were harvested and TIL isolated. TIL were stained for CD45.1 FITC, CD45.2 PerCPcy5.5, CD3 PE, and CD8 APC and analyzed by flow cytometry.
(PDF)

S2 Fig Increased tumor-specific response in bystander TIL after PV-10 injection in bilateral M05 Model. Mice received 3x10^5^ M05 cells SC in both flanks on Day 0. On Day 10, mice received 50 μl PV-10 or PBS IL in the left flank. Mice received anti-PD-1, anti PD-L1, or NrIgG on Days 10 and 13. On Day 17, the uninjected bystander lesion was resected and TIL were isolated. TIL were co-cultured with M05 or irrelevant MC38 tumor cells for 48 hours and supernatants were collected. IFN-gamma was measured by ELISA.
(PDF)

S3 Fig Depletion of T cells subsets. Mice received 3x10^5^ M05 tumor cells SC on a single flank on Day 0, and were given 300 μg IP of (A) NrIgG control antibodies, (B) 2.43 antibody to deplete CD8^+^ T cells, (C) GK1.5 antibody to deplete CD4^+^ T cells, or (D) PC61 antibody to deplete CD25^+^ Tregs. Antibodies were given twice per week until the completion of the experiment. Cell depletion was verified.
(PDF)

S4 Fig Combination treatment with checkpoint blockade and IL PV-10 leads to a delay in tumor growth in B16 tumor-bearing mice. (A) Mice received 1x10^5^ B16 tumor cells SC on Day 0. On Day 10, mice received a single injection of 50 μl PV-10 IL in combination with control IgG antibodies (NIgG) or in combination with anti-CTLA-4, anti-PD1, or anti-PD-L1 antibody IP. Antibody was injected twice per week and tumor was measured until the endpoint was reached.
(PDF)
