# Supplementary figures and images for: T cell mediated immunity after combination therapy with intralesional PV-10 and blockade of the PD-1/PD-L1 pathway in a murine melanoma model
Source: PLoS One. 2018 Apr 25;13(4):e0196033. doi: 10.1371/journal.pone.0196033 (PMC5918896; doi:10.1371/journal.pone.0196033)

**Fig S1**

**A**

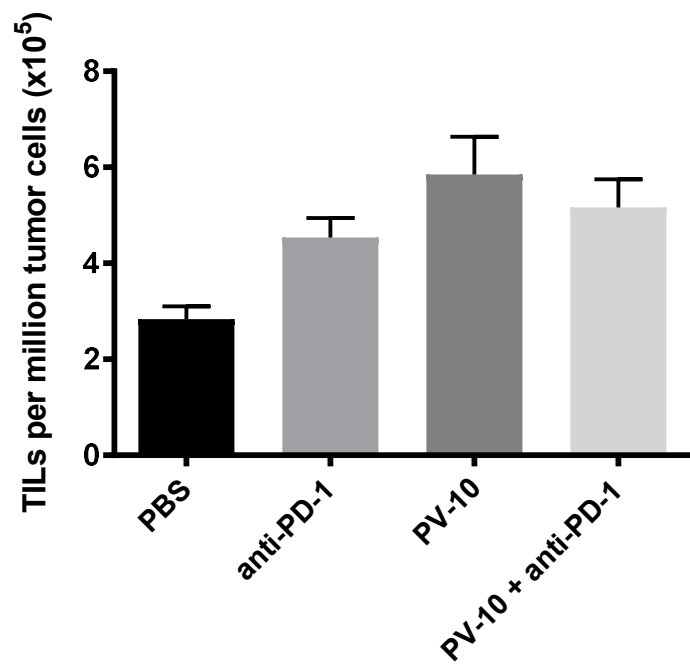

**B**

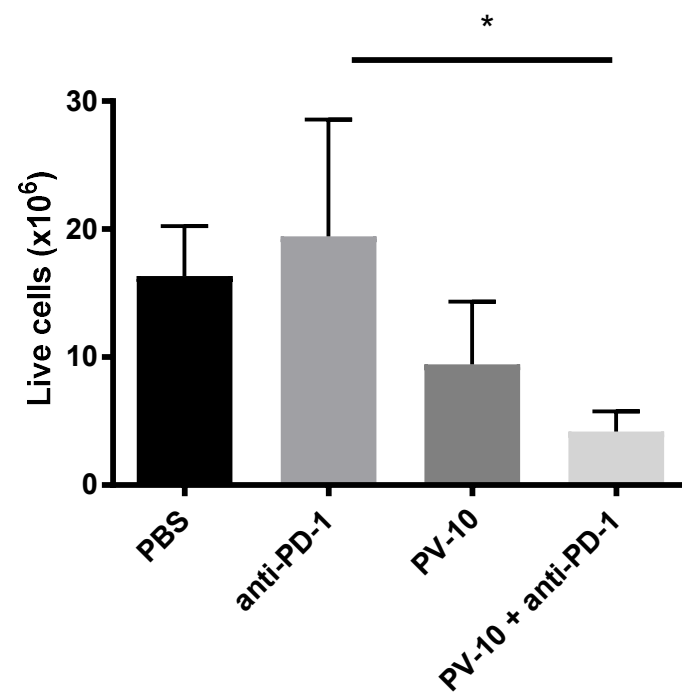

Supplement: S1 Fig — Mice received 3x10^5^ M05 tumor cells SC on Day 0. On Day 13, mice were injected IL with 50 μl PV-10 or PBS, 2.5x10^5^ violet-labelled CD45.1 OT-I T cells i.v., and 300 ug PD-1 IP. On Day 17, tumors were harvested and TIL isolated. TIL were stained for CD45.1 FITC, CD45.2 PerCPcy5.5, CD3 PE, and CD8 APC and analyzed by flow cytometry. (PDF) [file pone.0196033.s001.pdf]

**Fig S2**

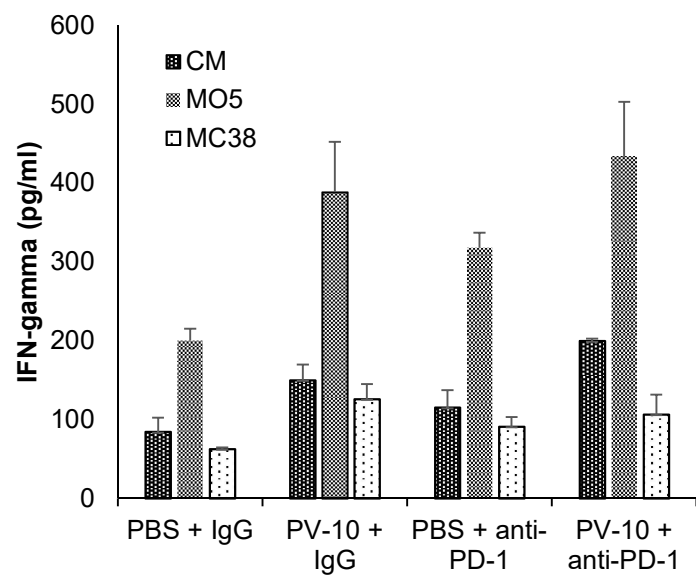

Supplement: S2 Fig — Mice received 3x10^5^ M05 cells SC in both flanks on Day 0. On Day 10, mice received 50 μl PV-10 or PBS IL in the left flank. Mice received anti-PD-1, anti PD-L1, or NrIgG on Days 10 and 13. On Day 17, the uninjected bystander lesion was resected and TIL were isolated. TIL were co-cultured with M05 or irrelevant MC38 tumor cells for 48 hours and supernatants were collected. IFN-gamma was measured by ELISA. (PDF) [file pone.0196033.s002.pdf]

Fig S3

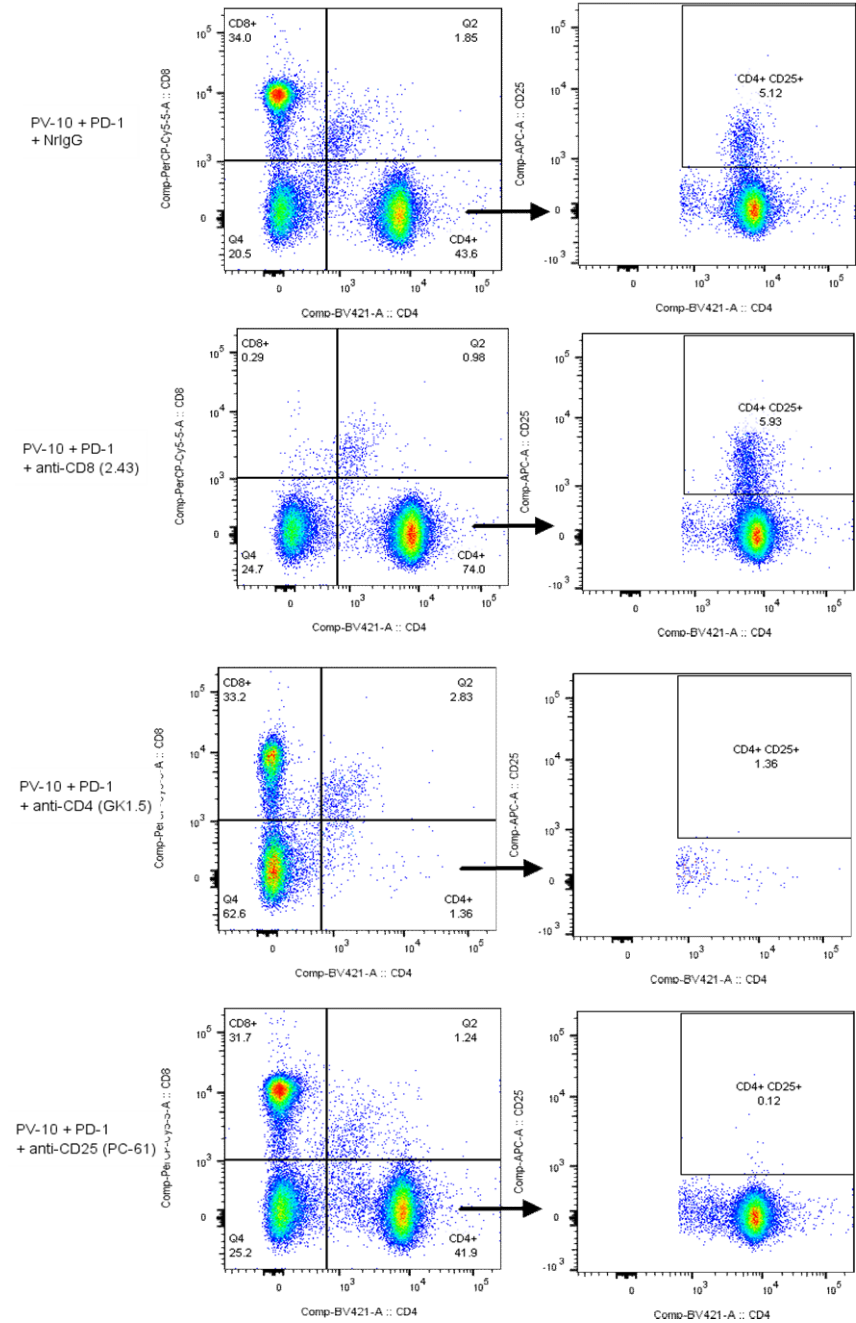

Supplement: S3 Fig — Mice received 3x10^5^ M05 tumor cells SC on a single flank on Day 0, and were given 300 μg IP of (A) NrIgG control antibodies, (B) 2.43 antibody to deplete CD8+ T cells, (C) GK1.5 antibody to deplete CD4+ T cells, or (D) PC61 antibody to deplete CD25+ Tregs. Antibodies were given twice per week until the completion of the experiment. Cell depletion was verified. (PDF) [file pone.0196033.s003.pdf]

Fig S4

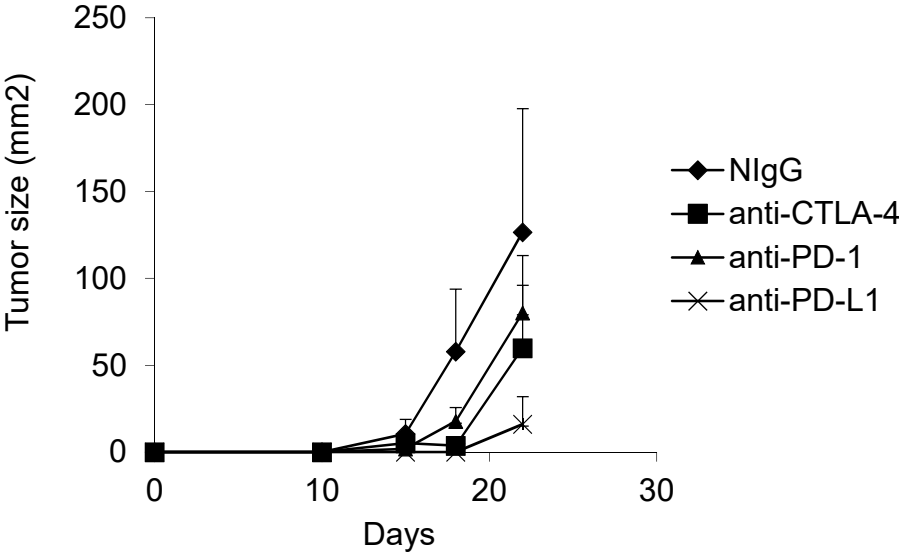

Supplement: S4 Fig — (A) Mice received 1x10^5^ B16 tumor cells SC on Day 0. On Day 10, mice received a single injection of 50 μl PV-10 IL in combination with control IgG antibodies (NIgG) or in combination with anti-CTLA-4, anti-PD1, or anti-PD-L1 antibody IP. Antibody was injected twice per week and tumor was measured until the endpoint was reached. (PDF) [file pone.0196033.s004.pdf]
